# Orthorexic eating behavior and body dissatisfaction in a sample of young females

**DOI:** 10.1007/s40519-020-00986-5

**Published:** 2020-08-15

**Authors:** Friederike Barthels, Julia Kisser, Reinhard Pietrowsky

**Affiliations:** Department of Clinical Psychology, Institute of Experimental Psychology, Heinrich Heine University Düsseldorf, Universitätsstraße 1, 40225 Düsseldorf, Germany

**Keywords:** Orthorexia nervosa, Orthorexic eating behavior, Eating disorders, Body image, Body dissatisfaction, Self-acceptance

## Abstract

**Purpose:**

To analyze body dissatisfaction in relation to orthorexic eating behavior in a sample of young females to further investigate characteristic features of orthorexic eating behavior and its association with other eating disorders.

**Methods:**

*N* = 197 young females (age: *M* = 29.59, SD = 10.85 years) completed an online survey with the following questionnaires: the Düsseldorf Orthorexia Scale to measure orthorexic eating behavior, the Eating Disorder Inventory-2 (EDI-2), measuring psychopathological aspects of disordered eating behavior, the Dresden Body Image Questionnaire (DKB-35) to measure five components of body image, and the Body Shape Questionnaire (BSQ), measuring body dissatisfaction.

**Results:**

In the total sample, Pearson correlations revealed that orthorexic eating behavior was positively associated with drive for thinness and body dissatisfaction. An independent samples *t*-Test revealed that females with elevated levels of orthorexic eating behavior (*n* = 35) displayed higher levels of drive for thinness and body dissatisfaction and lower levels of self-acceptance in comparison to a randomized sample from the remaining females with low levels of orthorexic eating behavior (*n* = 35). According to a multiple regression analysis, drive for thinness and body dissatisfaction measured by the BSQ served as positive predictors for orthorexic eating behavior, whereas bulimia and body dissatisfaction measured by the EDI-2 served as negative predictors.

**Conclusions:**

Results reveal that orthorexic eating behavior is more closely related to psychopathological aspects of other eating disorders than previously assumed. Body dissatisfaction as another major feature of orthorexia nervosa should be taken into account in future studies.

**Level of evidence:**

III, case–control analytic study.

## Introduction

When Bratman defined orthorexic eating behavior, he claimed the focus on the quality of food to be the feature that differentiated between orthorexic individuals and individuals with anorexia nervosa or bulimia nervosa who, he supposed, mainly focused on the quantity of food [1]. Consequently, authors inferred that the absence of body dissatisfaction and the desire to be thin served as distinguishing features of orthorexia nervosa from other eating disorders and included these aspects in definitions of orthorexia nervosa and proposals for diagnostic criteria or even stated that orthorexic individuals might have a positive body image (for a review of diagnostic criteria see [2]). However, a recent review [3] reports both, studies that support and studies that reject the hypothesis of orthorexic eating behavior being associated with body dissatisfaction. As a common limitation of these studies the authors mention the frequent usage of questionnaires with low psychometric properties.

The present study was designed to investigate the relation between orthorexic eating behavior and body dissatisfaction in a sample of young females, using the Düsseldorf Orthorexia Scale (DOS), a questionnaire which is reported to have good psychometric properties [4]. The study population was chosen because young age and female gender are considered to be potential risk factors for developing body dissatisfaction and eating disorders in general [5]. In this study we used both, questionnaires to assess psychopathological aspects of disordered eating behavior and body dissatisfaction [6, 7] as well as a questionnaire measuring five components of body image, with high scores indicating a positive relation to one’s body [8].

Hence, aims of the study were (1) to compute correlations between orthorexic eating behavior and positive and negative components of body image in a large sample of young females, (2) to conduct a multiple linear regression analysis to reveal if these variables are able to predict orthorexic eating behavior and (3) to compare a subgroup with elevated levels of orthorexic eating behavior to a subgroup with low levels of orthorexic eating behavior in terms of their relation to their body. We hypothesized correlations between orthorexic eating behavior and body dissatisfaction and differences between the subgroups regarding their relation to their body.

## Methods

### Sample

The total sample consisted of *N* = 197 predominantly young females (age: *M* = 29.59, *SD* = 10.85 years, BMI: *M* = 22.59, *SD* = 4.71 kg/m^2^; 43.7% students, 45.2% employed; 52.3% high school graduates) with a mean DOS score of *M* = 17.59 (*SD* = 5.52). Using the proposed cut-off-score for orthorexic eating behavior of 30 points [4] would have resulted in a sample too small to allow further comparisons (*n* = 7, 3.6% of the total sample). Using the proposed cut-off score of 25 points, supposed to indicate individuals being at risk to develop orthorexic eating behavior, would have resulted in a sample too small to achieve reliable results (*n* = 26, 13.2%) as well. Therefore, a cut-off point of 23 was chosen to obtain a good balance between elevated levels of orthorexic eating behavior and a sample large enough to allow reliable comparisons. The resulting sample consisted of *n* = 35 (17.8% of the total sample) females with a mean DOS score of *M* = 26.83 (*SD* = 3.55), called orthorexia group (OG). The control group (CG) was randomly drawn from the remaining sample, resulting in a group of *n *= 35 females with a mean DOS score of *M* = 14.63 (*SD* = 2.98). The groups did not differ regarding age (*M*_OG_ = 28.23, *SD*_OG_ = 11.19 years vs. *M*_CG_ = 29.77, *SD*_CG_ = 10.71; *t*(68) = 0.589, *p* = 0.558) nor BMI (*M*_OG_ = 22.61, *SD*_OG_ = 4.18 kg/m^2^ vs. *M*_CG_ = 22.87, *SD*_CG_ = 4.74 kg/m^2^; *t*(66) = 0.240, *p* = 0.811).

### Measures

All questionnaires were presented online. Data was collected anonymously without recording IP addresses. Participants were recruited via bulletin boards and social networks. They were informed that their participation was voluntary and anonymous and that their data were handled according to privacy policy. Furthermore, participants were informed that they could cancel the survey any time by not completing the questionnaire or by not sending their data using the send button. By sending their data, they agreed to participate in the study.

To assess orthorexic eating behavior, the Düsseldorf Orthorexia Scale [4] was used. It consists of 10 items to be rated on a 4-point scale ranging from *does not apply to me* (1) to *applies to me* (4), with high scores indicating high levels of orthorexic eating behavior. In this sample, Cronbach’s *α* was = 0.863.

To assess pathological characteristics associated with anorexic and bulimic eating behavior, the three subscales *drive for thinness*, *bulimia* and *body dissatisfaction* of the Eating Disorder Inventory-2 (EDI-2, German translation [6]) were used, consisting of 23 items to be rated on a 6-point scale from *never* (1) to *always* (6). In this sample, Cronbach’s *α* was = 0.897 for the subscale drive for thinness, *α* = 0.875 for the subscale bulimia and *α* = 0.913 for the subscale body dissatisfaction.

For the multidimensional assessment of body image with high mean scores indicating a positive relation to one’s body, the Dresden Body Image Questionnaire (DKB-35, [8]) was used. It consists of 35 items to be rated on a 5-point scale from *not at all* (1) to *totally* (5). Cronbach’s *α* of the five subscales was *α* = 0.884 for *vitality*, *α* = 0.919 for *self*-*acceptance, α* = 0.833 for *body contact*, *α* = 0.924 for *sexual satisfaction* and *α* = 0.784 for *self*-*aggrandisement*.

To assess dissatisfaction with one’s body, the Body Shape Questionnaire (BSQ, German translation [7]) was used. It consists of 34 items to be rated on a 6-point scale from *never* (1) to *always* (6). A high sum score indicates a high dissatisfaction with one’s body. In this sample, Cronbach’s *α* was = 0.974.

### Design and analysis

All analyses were conducted with IBM SPSS Statistics 26 for Mac OS. Sum scores and subscale scores for the DOS, EDI-2 and BSQ were calculated according to the instructions in the manuals. According to the manual of the DKB-35, subscale means were calculated. Regarding descriptive data, means (*M*), standard deviations (*SD*), absolute and relative frequencies are reported. In the total sample, Pearson correlations were computed between the DOS, the sum scores and the subscales of the used questionnaires. Additionally, partial correlations with BMI and age as control variables were computed. A multiple linear regression analysis using the enter-method was computed with EDI-2 and DKB-35 subscales and BSQ sum score as predictor variables and DOS sum score as outcome variable. Furthermore, independent *t*-tests were used to compare the mean scores of the EDI-2, DKB-35 and the BSQ between the OG and the CG. Cohen’s *d* was used to assess effect size. For all analyses, an *α* level of .05 was used. Sample sizes may vary due to missing values.

## Results

### Correlations and regression analysis in the total sample

The DOS was most strongly associated with the EDI-2 subscale drive for thinness and the BSQ sum score (see Table 1). The highest negative correlation could be observed between the DOS and the DKB-35 subscale self-acceptance. BMI and age as covariates in a partial correlation analysis changed the results only marginally.

**Table 1:** Bivariate and partial correlations (with BMI and age as control variables) of the DOS with subscales and total sum scores of EDI, DKB-35 and BSQ in a sample of young females (*N* = 197)

Scale	Bivariate correlations			Partial correlations		
	*N*	*r*	*p*	*df*	*r*	*p*
EDI-2						
Drive for thinness	195	0.65	< 0.001	140	0.70	< 0.001
Bulimia	195	0.31	< 0.001	140	0.28	< 0.001
Body dissatisfaction	192	0.34	< 0.001	140	0.42	< 0.001
DKB-35						
Vitality	190	− 0.09	0.205	140	− 0.06	0.493
Self-acceptance	188	− 0.44	< 0.001	140	− 0.48	< 0.001
Body contact	194	− 0.22	0.002	140	− 0.28	< 0.001
Sexual satisfaction	190	− 0.17	0.016	140	− 0.20	0.018
Self-aggrandisement	189	− 0.05	0.516	140	− 0.05	0.531
BSQ	177	0.61	< 0.001	140	0.64	< 0.001

*DOS* Düsseldorf Orthorexia Scale, *EDI-2* Eating Disorder Inventory-2, *DKB-35* Dresden Body Image Questionnaire, *BSQ* Body Shape Questionnaire

The regression analysis revealed that drive for thinness (*β* = 0.70, *t*(187) = 5.77, *p* < 0.001), bulimia (*β* = –0.26, *t*(187) = – 3.50, *p* = 0.001), body dissatisfaction (*β* = –0 .29, *t*(187) = – 2.80, *p* = 0.006) and BSQ sum score (*β* = 0.36, *t*(187) = 2.62, *p* = 0.010) significantly predict orthorexic eating behavior (*F*(9,154) = 19.60, *p* < 0.001, *R*^2^ = 0.55, *R*_adjusted_^2^ = 0.52).

### Group comparisons

The OG displayed significantly higher scores on the EDI-2 subscales drive for thinness and body dissatisfaction (see Table 2) than the CG. The OG also displayed higher BSQ sum scores. Furthermore, the OG displayed significantly lower scores on the DKB-35 subscale self-acceptance and sexual satisfaction than the CG. The groups did not differ on the other subscales.

**Table 2:** Mean scores (*M*) and standard deviations (*SD*) for the sample with elevated DOS scores (OG) and the control group (CG), including test statistics and effect sizes

Scale	OG			CG			*t* test		Effect size
	*M*	*SD*	*n*	*M*	*SD*	*n*	*t*(*df*)	*p*	*d*
DOS	26.83	3.55	35	14.63	2.98	35	− 15.56(68)	< 0.001	3.72
EDI-2									
Drive for thinness	30.85	7.79	34	17.97	7.31	35	− 7.09(67)	< 0.001	1.71
Body dissatisfaction	37.41	9.72	34	28.39	11.88	33	− 3.40(65)	< 0.001	0.83
Bulimia	13.71	5.82	34	11.21	4.61	34	− 1.96(66)	0.054	–
DKB-35									
Vitality	3.42	0.72	32	3.44	0.61	35	0.129(65)	0.898	–
Self-acceptance	2.52	0.91	34	3.49	0.93	32	4.27(64)	< 0.001	1.05
Body contact	3.74	0.82	35	4.03	0.81	34	1.48(67)	0.143	–
Sexual satisfaction	3.44	0.95	34	3.92	0.86	34	2.16(66)	0.034	0.53
Self-aggrandisement	3.05	0.77	33	3.11	0.72	34	0.360(65)	0.720	–
BSQ	137.16	30.36	31	78.03	32.20	31	− 7.44(60)	< 0.001	1.89

*DOS* Düsseldorf Orthorexia Scale, *EDI-2* Eating Disorder Inventory-2, *DKB-35* Dresden Body Image Questionnaire, *BSQ* Body Shape Questionnaire

## Discussion

The results revealed a moderate correlation of orthorexic eating behavior with body dissatisfaction measured by the EDI-2 subscale body dissatisfaction and a strong correlation with body dissatisfaction measured by the BSQ in a sample of young females. In addition, orthorexic eating behavior also correlates with drive for thinness and low self-acceptance. Entering these variables in a regression analysis revealed that drive for thinness and body dissatisfaction measured by the BSQ predict higher values of orthorexic eating behavior, whereas the EDI-2 subscales bulimia and body dissatisfaction predict lower values of the DOS, with 50% of explained variance in total. Hence, within the constructs of body dissatisfaction and bulimia that the EDI-2 captures, without the variances that the subscale drive for thinness already accounts for, there seem to be additional aspects that predict lower levels of orthorexic eating behavior. Future studies should investigate these aspects in detail, since they could potentially reveal which features differentiate orthorexic from anorexic eating behavior.

Group comparisons revealed that young females with elevated levels of orthorexic eating behavior displayed higher levels of drive for thinness and body dissatisfaction in comparison to a control group with low levels of orthorexic eating behavior. Furthermore, these females also displayed less self-acceptance. Interestingly, these differences reached statistical significance, although mean DOS scores of the orthorexia group did not exceed the suggested cut-off score for orthorexic eating behavior. Hence, even young females with slightly elevated levels of orthorexic eating behavior might be prone to develop psychopathological features that are characteristic of anorexia nervosa and bulimia nervosa, underlining the potential of orthorexia nervosa to be accompanied by more severe symptoms of disordered eating behavior. This conclusion is further emphasized when comparing the mean scores of drive for thinness, body dissatisfaction and BSQ sum score of our sample to normative data of healthy females [6, 7]. For the subscale drive for thinness, mean scores of the OG fall within the 99th percentile (CG: 60th percentile), for the subscale body dissatisfaction, mean scores fall within the 75th percentile (CG: 50th percentile) and for the BSQ sum score, mean scores fall within the 99 percentile (CG: 75 percentile). Furthermore, these findings indicate that orthorexic eating behavior might be more similar to other eating disorders, especially to anorexia nervosa, than previously assumed [1]. In the literature, the relation of orthorexic and anorexic eating behavior is thoroughly discussed, presenting both, arguments for and against the assumption of orthorexia nervosa either being a distinct eating disorder or a variant of anorexia nervosa [9]. In line with other studies (e.g., [10]) our results support the hypothesis of orthorexic eating behavior sharing psychopathological features (in this case: body dissatisfaction) characteristic of other eating disorders, especially anorexia nervosa, suggesting a close relation between these disorders.

### Limitations

The following limitations must be taken into account while interpreting the results of this study. Although DOS scores of the OG are elevated, they do not exceed the preliminary cut-off score for orthorexic eating behavior. Therefore, this group can only be considered to be at risk of developing orthorexic eating behavior. However, this aspect might also indicate that even in a risk group, body dissatisfaction, as a psychopathological feature of disordered eating behavior, is present. Furthermore, our sample consists solely of young females, hence, the results cannot be generalized to males or mixed samples or to older individuals. Another concern might be that the DOS does not measure orthorexic eating behavior but other aspects of disordered eating behavior. Although studies suggest that construct validity of the DOS is promising [e.g., 11], more data is needed to rule out the possibility that the DOS erroneously captures aspects not only indicative of orthorexic eating behavior.

## Conclusion

This brief report reveals that orthorexic eating behavior is more closely related to psychopathological aspects of the other eating disorders than previously thought. Body dissatisfaction as another major feature of orthorexia nervosa should be taken into account in further studies and also in diagnostic criteria as well as in the treatment of orthorexic eating behavior. The aspects of body dissatisfaction that seem to predict lower values of orthorexic eating behavior should be investigated in future studies as well.

### What is already known on this subject?

While some studies suggest that orthorexic eating behavior is not associated with body dissatisfaction, other studies reveal a correlation between these two variables.

### What does this study add?

The results indicate correlations between orthorexic eating behavior and body dissatisfaction in young females and higher levels of body dissatisfaction in young females with elevated levels of orthorexic eating behavior.

## Data Availability

Upon request, original data is available from the corresponding author.
