# Correction to: Orthorexic eating behavior and body dissatisfaction in a sample of young females

**DOI:** 10.1007/s40519-021-01219-z

**Published:** 2021-06-04

**Authors:** Friederike Barthels, Julia Kisser, Reinhard Pietrowsky

**Affiliations:** Department of Clinical Psychology, Institute of Experimental Psychology, Heinrich Heine University Düsseldorf, Universitätsstraße 1, 40225 Düsseldorf, Germany

## Correction to: Eating and Weight Disorders – Studies on Anorexia, Bulimia and Obesity 10.1007/s40519-020-00986-5

The article “Orthorexic eating behavior and body dissatisfaction in a sample of young females”, written by Friederike Barthels, Julia Kisser and Reinhard Pietrowsky, was originally published electronically on the publisher’s internet portal on 15 August 2020 without open access. With the author(s)’ decision to opt for Open Choice, the copyright of the article changed on 18 May 2021 to © The Author(s) 2021 and the article is forthwith distributed under a Creative Commons Attribution 4.0 International License, which permits use, sharing, adaptation, distribution and reproduction in any medium or format, as long as you give appropriate credit to the original author(s) and the source, provide a link to the Creative Commons licence, and indicate if changes were made. The images or other third party material in this article is included in the article’s Creative Commons licence, unless indicated otherwise in a credit line to the material. If material is not included in the article’s Creative Commons licence and your intended use is not permitted by statutory regulation or exceeds the permitted use, you will need to obtain permission directly from the copyright holder. To view a copy of this licence, visit http://creativecommons.org/licenses/by/4.0. Open access funding enabled and organized by Projekt DEAL.

The original article has been corrected.

